# Clinical characteristics and survival outcomes in patients with ovarian strumal carcinoid

**DOI:** 10.1186/s12885-022-10167-5

**Published:** 2022-10-24

**Authors:** Sijian Li, Xiaoxue Wang, Xiaolong Sui, Xinyue Zhang, Min Yin, Jiaxin Yang

**Affiliations:** 1 National Clinical Research Center for Obstetric and Gynecologic Diseases, Department of Obstetrics and Gynecology, Peking Union Medical College Hospital, Chinese Academy of Medical Sciences, Peking Union Medical College, Beijing, People’s Republic of China; 2 Department of Pathology, The Affiliated Yantai Yuhuangding Hospital of Qingdao University, Yantai, Shandong People’s Republic of China

**Keywords:** Strumal carcinoid, Ovarian carcinoid, Clinical characteristics, Treatment, Survival outcomes

## Abstract

**Background:**

Ovarian strumal carcinoid is an extremely rare ovarian malignant tumor with limited data on clinical characteristics and survival outcomes.

**Methods:**

A retrospective study of 119 patients was conducted, including 98 cases identified from literature review, and their clinical characteristics were investigated. The overall survival (OS), disease-specific survival (DSS), recurrence-free survival (RFS), and potential prognostic factors of these patients were also evaluated.

**Results:**

Lesions of 115 cases were confined to the ovarian while four patients presented with extra-ovarian disease upon initial diagnosis. Surgical treatment options performed in this cohort varied, 5.0% received ovarian cystectomy, 36.1% received unilateral salpingo-oophorectomy (USO), 7.6% received bilateral salpingo-oophorectomy (BSO), 42.0% received hysterectomy with BSO, and 8.4% underwent debulking surgery. Moreover, one patient did not undergo any surgery. No postoperative adjuvant therapy was administered in 89.9% patients, while 7.6% and 2.5% received adjuvant radiotherapy and chemotherapy, of which two patients received combined radiation and chemotherapy. At the final follow-up, 89.1% patients showed no evidence of the disease, and 5.0% were alive with the disease. Only seven deaths occurred, with two attributed to the tumor. The 5-year, 10-year, and 20-year OS rates were 96.0%, 85.0%, and 85.0%, respectively, with a 15-year recurrence rate of 4.4%. The 5-year and 20-year DSS rate were 98.5% and 95.9%. Multivariate Cox regression showed age ≥ 55 years was the only risk factor associated with the OS (*P* = 0.014, OR 7.988; 95% CI 1.519 – 42.004). However, the univariate and multivariate Cox regression showed no potential risk factor for RFS and DSS.

**Conclusion:**

Patients with ovarian strumal carcinoid have an excellent prognosis irrespective of the surgical option. Conservative surgery especially USO with individualized adjuvant therapy is recommended.

**Supplementary Information:**

The online version contains supplementary material available at 10.1186/s12885-022-10167-5.

## Introduction

Primary ovarian carcinoid tumor (POCT), a type of neuroendocrine tumor of the female genital tract, is extremely rare and only accounts for approximately 1% of the carcinoid tumors and less than 0.1% of malignant ovarian tumors [1–3]. POCT can be divided into four histological subtypes: insular, trabecular, strumal, or mucinous carcinoid. Strumal carcinoid is defined as an intimate mixture of thyroid tissue and carcinoid components and was first described in the 1970s [4]. There have only been about 100 cases reported after more than five decades [3]. Characterization of the management of this rarity is mainly accumulated through case report, and treatment strategies have varied greatly, with some researchers arguing for comprehensive staging surgery while others advocated unilateral salpingo-oophorectomy [2,5–9]. Postoperative adjuvant therapies, such as chemotherapy and radiotherapy, have also been reported [10,11], but with uncertain benefit.

Moreover, few studies with a large sample size have evaluated the survival outcomes in this population. Only one larger series of 50 cases analyzed clinical features of this disease in the 1980s, but it did not further investigate potential survival risk factors [7]. In 2020, Theurer et al. reported 13 cases of strumal carcinoids focusing on their immunohistochemical and molecular characteristics [4]. A study including 588 patients showed excellent survival outcomes, but this study included ovarian carcinoid tumors of other pathological subtypes and did not evaluate tumor-related factors affecting death in multivariate analysis [12]. The detailed information on clinical characteristics and prognosis, especially prognostic predictors, needs to be explored in greater depth.

To investigate the clinical characteristics and outcomes of patients with ovarian strumal carcinoid, we conducted a retrospective study of 119 patients, including 21 patients diagnosed in Peking Union Medical College Hospital and 98 cases reviewed in published research. The overall survival (OS), disease-specific survival (DSS), recurrence-free survival (RFS), and potential prognostic factors of these patients were further evaluated.

## Methods

We firstly retrospectively evaluated 21 patients with ovarian strumal carcinoid diagnosed in our hospital. Then a systematic literature review was conducted to collect all available cases published in English in PubMed, Embase, and Scopus from 1970 to 2021 using the following keywords: ‘‘ovarian strumal carcinoid’’, ‘‘strumal carcinoid’’, ‘‘metastatic ovarian strumal carcinoid’’, ‘‘strumal carcinoid arising in ovary’’, ‘‘strumal carcinoid tumor of the ovary’’, and ‘‘struma ovarii’’. We also reviewed possible relevant references cited in these articles. Patients with pure struma ovarii, malignant struma ovarii, metastatic struma ovarii, ovarian carcinoid tumor in other subtypes (insular and trabecular), or lack of demographic data, treatment methods, or follow-up results were excluded. Supplementary Figure S1 shows the detailed inclusion process of this study. We eventually generated a database including 119 cases of ovarian strumal carcinoid documenting the demographic characteristics, survival outcomes, and clinical, pathological, and treatment features. In this database, 21 patients were treated in our hospital, while the other 98 cases were selected from 46 reviewed articles.

Several potential independent prognostic factors were examined, including age at diagnosis (< 55 or ≥ 55 years. The cutoff was based on the American Joint Committee on Cancer (AJCC) staging system for differentiated thyroid cancers and researches on malignant struma ovarii [13,14]), tumor components (containing only teratoma components or including non-teratoma components, such as cystadenoma), tumor size (< 10 or ≥ 10 cm, the cutoff was selected as the mean mass size was 10 cm), surgical options (conservative surgery or radical surgery), and adjuvant therapy (with or without adjuvant therapy). In this study, conservative surgery was defined as ovarian cystectomy with or without appendectomy, USO with or without appendectomy, while radical surgery was defined as BSO with or without appendectomy, H/BSO with or without appendectomy, or debulking surgery, concerning the fertility preserving. Regular serum tumor makers (Neuron Specific Enolase (NSE), CA 19–9, and CA 125, etc.) combined with imaging examination were administered for follow-up in patients with strumal carcinoid from the initial treatment till final follow-up. Recurrence-free survival (RFS) was defined as the date from initial treatment intervention to confirmed tumor recurrence or metastasis. Overall survival (OS) was defined as the time from the date of initial diagnosis to death associated with any cause or final follow-up. Disease-specific survival (DSS) was defined as the time from the date of the initial diagnosis to death related to ovarian strumal carcinoid or final follow-up.

### Statistical analysis

Different types of variables were displayed in corresponding formations. Mean ± standard deviation (range) was used to describe continuous variables that were normally distributed, while all others were presented as medians and interquartile ranges (IQRs). Meanwhile, counts (percentages) were used to express discrete variables, and categorical variables were compared by the chi-squared test. The Kaplan–Meier method (log-rank test) was used to compare survival rates between subgroups and establish survival curves. Univariate analysis was performed to screen clinical prognostic factors for RFS, OS, and DSS. Factors with *p*-values < 0.10 were included in subsequent multivariate analysis using the Cox regression model to identify independent prognostic factors. A two-tailed *p*-value < 0.05 was considered significant. The statistical analysis was conducted with SPSS (Version 20.0; SPSS Inc., Chicago, IL, USA) or GraphPad Prism (Version 8.0) software.

## Results

### Results of 21 cases in Peking Union Medical College Hospital

#### Demographic data and clinical characteristics

The mean age of the patients was 45.6 years, with a median age of 46.0 years (range: 28–65). Most patients (19/21, 90.5%) presented with an asymptomatic pelvic mass or mild abdominal discomfort with pelvic mass. Only one patient experienced dysmenorrhea, and another patient presented with ascites. Elevated tumor markers were noted in only three cases. Among them, one had elevated serum CA 125, and another had increased NSE, while elevated CA 19–9, CA 125, and CEA were observed in the third patient. However, no patients showed constipation or carcinoid syndrome. The mean mass size was 6.9 cm, ranging from 2 to 18.4 cm (Table 1).

**Table 1:** The 21 cases of ovarian stromal carcinoid in Peking Union Medical College Hospital

Patients	Age (y)	Manifestations	Elevated tumor marker	Mass (carcinoid) size (cm)	Surgery	Ki-67 index	Adjuvant therapy	Result of follow-up
1	35	pelvic mass	N	5.8	LSO		N	NED at 9.25y
2	56	dysmenorrhea; menorrhagia	N	6.7	LH + BSO	< 1%	N	NED at 7.75y
3	32	pelvic mass	N	5.9	Ovarian cystectomy		N	NED at 6.83y
4	48	pelvic mass	N	3.3	LH + BSO		N	NED at 5.83y
5	47	pelvic mass	N	4.3	TAH + BSO	3%	N	NED at 3.16y
6	28	pelvic mass	N	5	RSO		N	NED at 2.42y
7	53	pelvic mass	N	6	LSO	1%	N	NED at 2.17y
8	42	pelvic mass	N	10	LH + BSO		N	NED at 3.5y
9	44	pelvic mass	N	7.2	RSO		N	NED at 9.6y
10	39	pelvic mass	N	5	LSO		N	NED at 11.75y
11	38	abdominal distention; ascites	N	7.9	LSO	2%	N	NED at 5.45y
12	30	pelvic mass	N	7	LSO	5%	N	NED at 10.1y, naturally conceived and delivered a full-term baby
13	46	pelvic mass	N	8.4	Ovarian cystectomy	5%	N	NED at 0.3y
14	46	pelvic mass	N	2	TAH + LSO (RSO for pelvic abscess previously)	5%	N	NED at 0.25y
15	65	pelvic mass	Y (CA 125 45.7U/ml)	5.9	LH + BSO + LN (pelvic and para-aortic) + appendectomy	< 1%	N	NED at 5.1y
16	47	pelvic mass	Y (NSE 19.2 ng/ml)	7.9 (1.3)	LH + BSO	1%	N	NED at 3.3y
17	64	abdominal pain, pelvic mass	Y (CA125 164.3U/ml, CA19-9 391.3U/ml, CEA 10.79 ng/ml)	18.4	LH + BSO	1%	N	NED at 1.3y, coexisted with ovarian adenocarcinoma
18	64	pelvic mass	N	8	TAH + RSO + omentectomy; LSO for teratoma previously		N	NED at 20y
19	30	pelvic mass	N	4 (0.25)	Ovarian cystectomy	2%	N	NED at 1.5y
20	48	abdominal distention	N	10 (0.2)	TAH + BSO + omentectomy + LN (pelvic and para-aortic) + appendectomy		Chemotherapy	NED at 25.4y
21	55	pelvic mass	N	7	LH + BSO	3%	N	NED at 0.4y

*Abbreviations*: *N* No, *Y* Yes, *L/RSO* Left/right salpingo-oophorectomy, *BSO* Bilateral salpingo-oophorectomy, *LH/TAH* Laparoscopic/total abdominal hysterectomy, *LN* Lymph nodes resection; *NED* No evidence of disease

#### Surgical treatment, pathological results, and postoperative adjuvant therapy

**Fig. 1:**
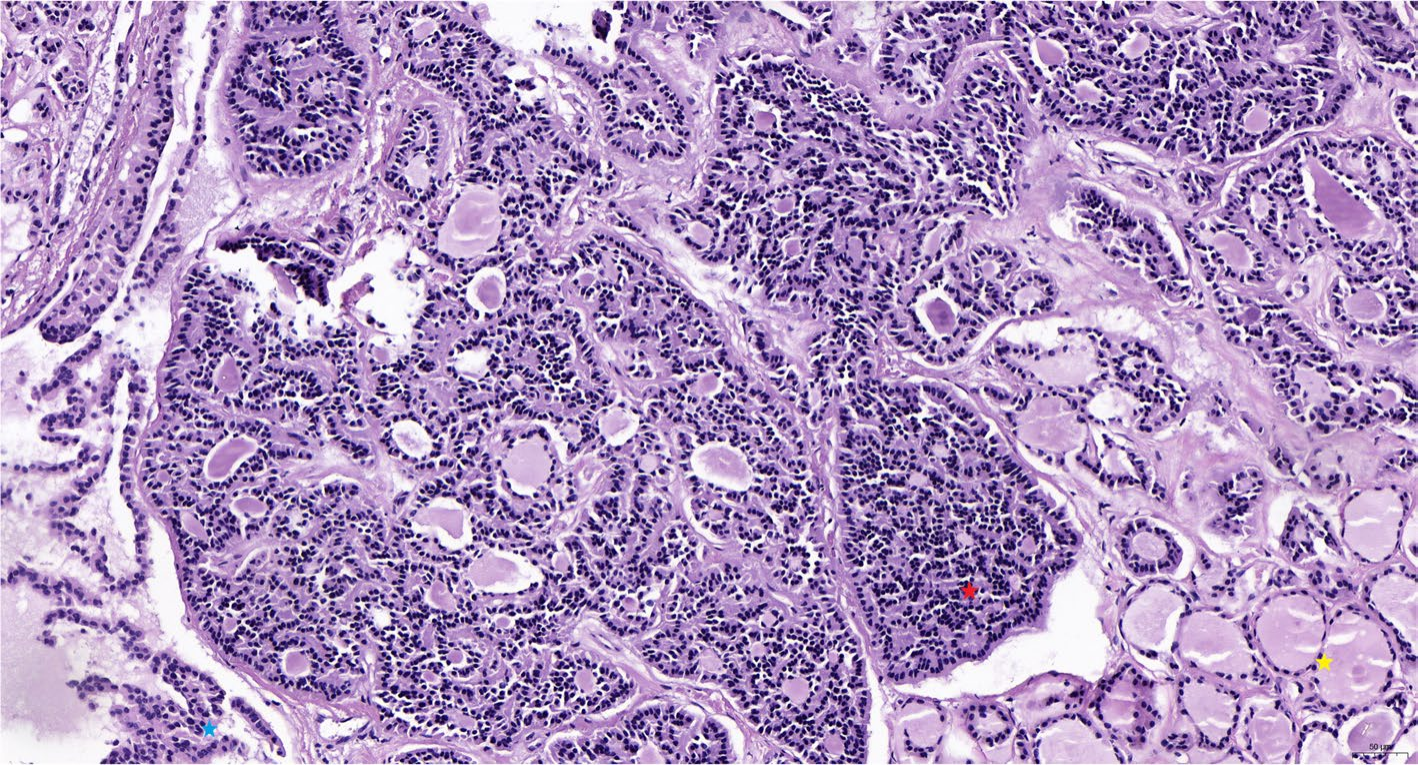
Pathological features of ovarian strumal carcinoid synchronous adenocarcinoma (yellow star, struma ovarii; red star, carcinoid component; blue star, adenocarcinoma component; case 17, HE staining, 200X)

Different surgical options were administrated in these patients, including ovarian cystectomy, unilateral salpingo-oophorectomy (USO), hysterectomy with bilateral salpingo-oophorectomy (H/BSO), and debulking surgery (H/BSO plus retroperitoneal lymph node resection and appendectomy). Seven patients underwent USO, and H/BSO was another commonly applied surgical method performed for another eight patients. Meanwhile, three patients received a less common surgery, ovarian cystectomy. Additionally, three patients underwent debulking surgery. Pathological examination showed that carcinoid coexisting with struma ovarii was the most common subtype (15/21, 71.4%), and the other four were strumal carcinoid synchronous with mature cystic teratoma component. However, one patient (case 17) was coexisted with ovarian adenocarcinoma (Fig. 1). Ki-67 proliferation index was available in 12 cases (57.1%), and none exceeded 5% (Table 1). Regardless of surgical strategy, most patients did not receive any postoperative adjuvant therapy, and only one patient (case 20) was administrated adjuvant chemotherapy (cisplatin plus cyclophosphamide for 6 cycles).

#### Results of follow-up

Serum tumor markers were measured combined with imaging screening to detect potential recurrent lesions every 3 to 6 months. All patients lived with no evidence of the disease during a median follow-up of 5.1 years (range 3 months – 25.4 years, with a mean follow-up time of 6.4 years), and none experienced recurrence.

### Literature review

This retrospective analysis included 119 patients, and the mean age was 49.1 years, with a median of 49.0 years (range 20 – 78) (Supplementary Table S1). Childbearing women were less affected as only 19.3% of patients were aged ≤ 35 years. The gross tumor size varied significantly in these patients, with the mean mass size 10.0 ± 5.1 cm, ranging from 1.0 to 26.0 cm. No patient presented with synchronous bilateral strumal carcinoid. At the time of initial diagnosis of ovarian strumal carcinoid, lesions in 115 cases (96.6%) were confined to the ovary, while extra-ovarian metastasis was only manifested in four cases (3.4%).

Patients with ovarian strumal carcinoid were generally asymptomatic or presented nonspecific discomfort. In 79 patients with reported clinical symptoms, 38.0% and 20.2% of them reporting pelvic mass or complaining abdomen pain/discomfort, respectively. Other common manifestations showing in descending order were constipation (15 cases, 19.0%), vaginal bleeding (7 cases, 8.9%), and hirsutism (5 cases, 4.2%). Hypoglycemia, carcinoid heart disease, nausea, diarrhea, and ascites were much less common compared to the primary complaints. It’s worth mentioning that some patients presented with two or more of the above-mentioned symptoms (Table S2). Ovarian strumal carcinoid was generally nonfunctional with only 18.5% presenting with endocrine function, among which there were 15 cases of constipation, five cases of hirsutism, and two cases of hyperinsulinism. However, carcinoid syndrome was extremely rare and found only in one patient (Table 2). Moreover, dysthyroidism was rarely seen in ovarian strumal carcinoid that only one patient presented hyperthyroidism and three manifested hypothyroidism.

**Table 2:** Clinical characteristics of patients with ovarian stromal carcinoid

Clinical characteristics	Number (Percentile)
Age (Mean/Median, y)	49.1 ± 13.7/49.0 (20–78)
Time of follow-up (Mean/Median, y)	5.5/3.0 (0.08–32)
Tumor size (*N* = 116)	10.0 ± 5.1 (1.0- 26.0)
Coexisted Carcinoid syndrome	1 (0.84%)
Functional struma	22 (18.5%)
Extra-ovarian metastasis	
Yes	4 (3.4%)
No	115 (96.6%)
Lymph node resection positive (*N* = 7)	0 (0%)
Metastatic site	
Liver	2
Bowel	2
Peritoneum	1
Omentum	1
Appendix	1
Recurrence	3 (2.6%)
Liver	1
Multiple bone and breast	1
Contralateral ovary and para-aortic lymph nodes	1
Treatment in recurrence	
No	1
RSO + para-aortic lymph node resection	1
Radiotherapy + chemotherapy	1

*Abbreviations*: *RSO* Right salpingo-oophorectomy

**Fig. 2:**
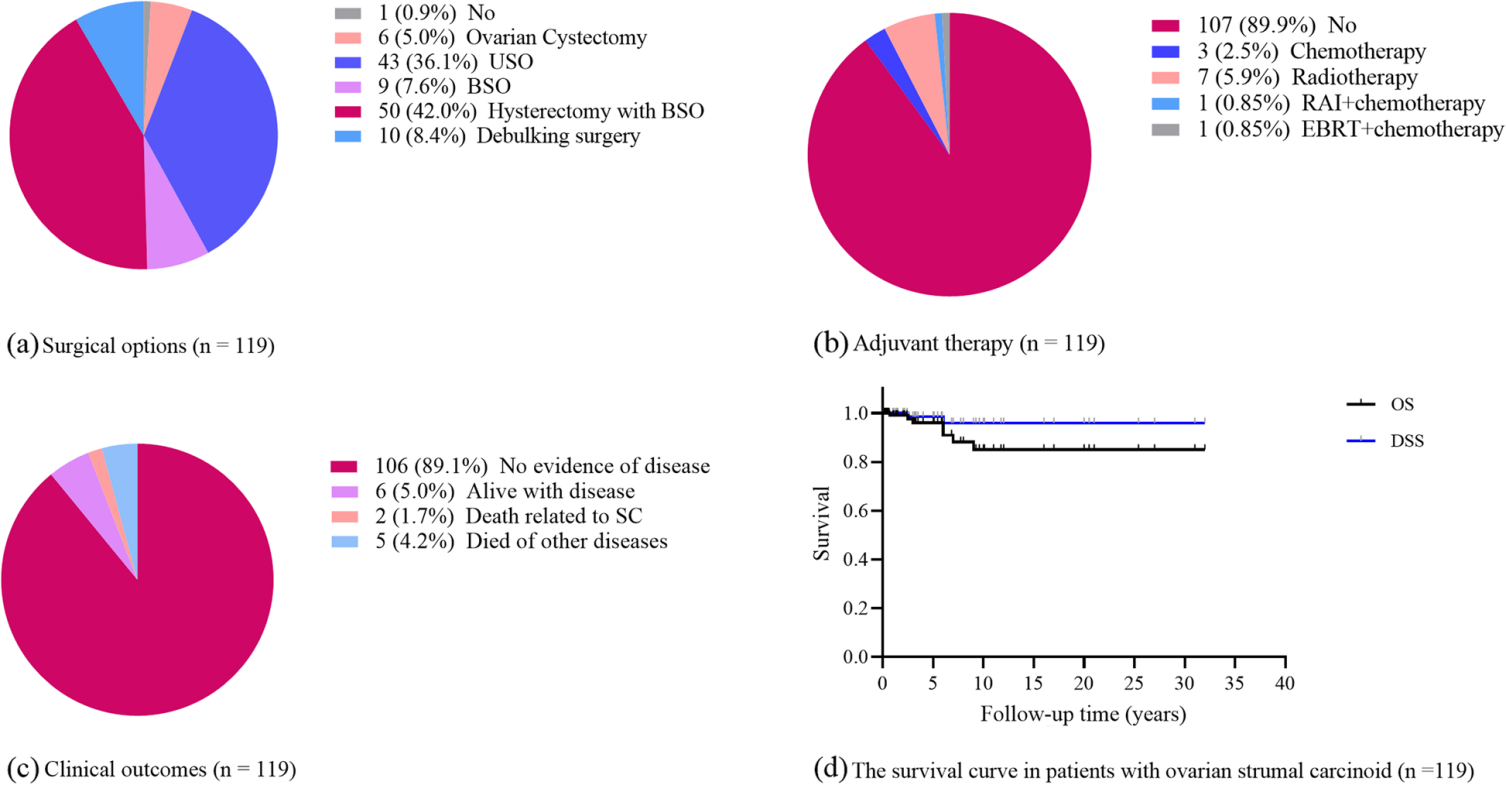
Treatment and clinical outcomes in patients with ovarian strumal carcinoid. **a** Surgical options, **b** adjuvant therapy, **c** clinical outcome, **d** overall survival and disease-specific survival curve for patients enrolled in our study

Pathological results showed that 91.5% of strumal carcinoids arose in struma ovarii or mature teratoma, and the other 22 cases (18.5%) contained non-teratoma components (cystadenoma, malignant struma ovarii, adenocarcinoma, etc.). Ki-67 proliferation index was available for 21 cases, and 13 (61.9%) had Ki-67 not exceeding 2%, and only two patients had Ki-67 over 5% (10% and 12%). Forty-nine patients (41.1%) received conservative surgery, and 58.0% underwent radical surgery with only one patient not receiving any surgery. Regarding specific surgical options, hysterectomy with BSO was the most common, wherein 42.0% of patients underwent this surgery, followed by USO (36.1%), debulking surgery (8.4%), BSO (7.6%), and ovarian cystectomy (5.0%). In addition, one patient who underwent ovarian cystectomy also experienced metastasectomy (liver and peritoneum lesions). Pelvic and para-aortic lymphadenectomy was applied in seven patients but none showed tumor involved. A total of 14 patients also underwent appendectomy, seven of them with H/BSO, five of them with USO, and each one in cystectomy and BSO. However, none of these patients had metastatic tumors of ovarian strumal carcinoid. After surgery, 89.9% of patients received no postoperative adjuvant therapy, 7.6% of them received radiotherapy, while 2.5% were administered chemotherapy, with two receiving combined radiotherapy and chemotherapy (Table 2 and Fig. 2a and b).

During the follow-up, four patients did not achieve complete remission, and four patients experienced recurrent disease within 10 months to 31 years. The recurrent sites in these patients were liver (two cases, in which one coexisted with peritoneal metastasis), bone and breast (one case), contralateral ovary, and para-aortic lymph nodes (one case). Surgery after recurrence was performed for two patients, in which the individual who had liver and peritoneal metastasis only received laparotomy exploration, and USO and lymph nodes resection was performed in the other. The other patient with liver metastasis merely underwent close follow-up with stable disease. The patient with bone and breast metastasis successively received chemotherapy and radiotherapy but remained insensitive. At the final follow-up, 89.1% (106) of these patients showed no evidence of disease, and 5.0% (6) were alive with disease. Only seven deaths occurred, with two attributed to ovarian strumal carcinoid (Fig. 2c). The other five patients died of rheumatic heart disease, auto accident, cerebrovascular accident, intestinal necrosis, and cirrhosis.

The mean follow-up time was 5.5 years, with a median follow-up time of 3.0 years. The 5-year, 10-year, and 20-year OS rates were 96.0%, 85.0%, and 85.0%, respectively, with a 15-year cumulative recurrence rate of 4.4%. The 5-year and 20-year DSS rate were 98.5% and 95.9%, respectively (Fig. 2d, Figure S2). Factors that potentially affect OS outcomes are summarized in Supplementary Table S3a, and univariate analysis showed that age (Age < 55y vs. Age ≥ 55 years; *P* = 0.003, Figure S3) and mass size (< 10 vs. ≥ 10 cm; *P* = 0.028) were significantly associated with poor overall prognosis. Age and mass size were used for further multivariable analysis, where an age ≥ 55 years (*P* = 0.014, OR 7.988; 95% CI 1.519 – 42.004) remained statistically significant. No statistically significant factors were found associated with RFS and DSS (Table S3b and S3c). However, the proportion of recurrence was 50% (1 in 2 patients) in those who had Ki-67 index higher than 10%, while the rate was 5.3% (1 in 19) in patients who had Ki-67 index less than 10% (*P* = 0.186).

## Discussion

Our study presents the largest cohort of ovarian strumal carcinoids focusing on clinical features and survival outcomes in this unique population. Patients with strumal carcinoid mostly manifested lesions confined to the unilateral ovary with nonspecific symptoms. The survival outcome was excellent regardless of therapeutic strategies or the presence of metastasis, and age over 55 years significantly impaired the OS.

The pathogenesis of ovarian strumal carcinoid remains unclear. Theurer et al. evaluated 13 cases strumal carcinoids but did not reveal any known pathogenic and relevant mutations identified commonly in thyroid carcinoma and neuroendocrine tumors (NET), suggesting that these tumors maybe genetically unrelated to follicular epithelial-derived thyroid tumors and potentially different than other commonly identified NET [4]. Strumal carcinoid usually affected women of perimenopausal age as the mean age was younger than that of patients with insular carcinoid [3]. However, still, nearly 20% of patients were younger than 35-years-old, so fertility-sparing options should not be disregarded. None of these patients presented bilateral strumal carcinoid, which could be an important point of differential diagnosis with carcinoid tumor metastatic to the ovary. Our results were consistent with Robboy et al. showing that carcinoid syndrome and hirsutism/masculinization were rare in patients with strumal carcinoid [7], but the rate of functional struma was higher, especially in the 15 cases of patients presenting with constipation. It prompts the consideration of this disease when patients present pelvic mass and constipation because the peptide-YY secreted by strumal carcinoid can inhibit gastrointestinal motility. However, hyperthyroidism related to strumal carcinoid or coexisting with primary thyroid cancer has never been reported in published cases, which suggested that rigorous surveillance of thyroid function or cancer screening may be omitted.

To the best of our knowledge, this is the first study characterizing the OS, RFS, and DSS rate in this population. Robboy et al. reported in 1980 that six death occurred, with only one patient death due to the tumor and 23 patients surviving for at least five years, and 15 patients surviving for at least ten years postoperatively in a series of 50 patients [7]. However, they did not evaluate potential risk factors affecting OS and RFS, and it was necessary to review the dozens of cases reported in the following decades with updated information on clinical outcomes in patients with strumal carcinoid. Nonetheless, almost all of them were case reports, and another case series containing 13 patients did not focus on survival [4]. Recently, Nasioudis et al. analyzed 588 patients with primary malignant ovarian carcinoid, and they found that patients with early-stage disease had excellent OS compared to those with advanced-stage (II-IV) disease (P < 0.001; 5-year OS rates were 95.4% and 53.1%, respectively) [12]. Nevertheless, they neither presented the 5-year OS rate in this overall cohort nor restricted their study to patients with strumal carcinoid, as patients with insular, trabecular, and mucinous carcinoid tumors were also included. Meanwhile, these factors related to survival were not further examined by multivariate analysis.

Our research extended these findings. The survival outcomes in this cohort were excellent with 5-year and 20-year OS at 96.0% and 85.0%, respectively, with a 20-year DSS rate of 95.9%. Based on the current evidence, a reasonable suggestion is that treatment administered to patients in this population should prioritize the importance of their quality of life and avoid unnecessary therapy. We found that age over 55 years was the only independent risk factor for OS but failed to predict DSS. Age > 55 years as a prognostic predictor has been validated in well-differentiated thyroid cancer and metastatic malignant struma ovarii [13,14]. This suggested that these three diseases might share something in common due to the thyroid component. Meanwhile, deaths occurring in this study were more commonly attributed to other complications, such as cirrhosis and cerebrovascular diseases, which more often affect older people [15]. This also prompts clinicians to pay attention to patients’ co-morbidities.

Previously, Robboy et al. stated the effectiveness of oophorectomy or salpingo-oophorectomy, but they did not clearly advocate for unilateral or bilateral salpingo-oophorectomy as the preferred option [7]. Nearly half of the patients in our cohort received conservative surgery, and USO/BSO was applied in 43.7% of patients. Meanwhile, surgical options were not associated with survival outcomes, and hysterectomy with BSO or more extended surgical procedures did not significantly improve the OS, RFS, and DSS. Although the six patients treated with ovarian cystectomy obtained NED at the final follow-up, one patient experienced recurrence 30 years later. Ovarian cystectomy may also lead to tumor residue, rupture, and intraoperative seeding, which is inconsistent with the principles of oncological surgery. Since no patients presented with bilateral strumal carcinoid, we suggest that USO should be the preferred surgical option in this population, and ovarian cystectomy may be preserved in patients who desire the preservation of fertility and have previously received contralateral oophorectomy. In patients with extra-ovarian metastatic disease, USO with metastasectomy may be more suitable to balance the prognosis and quality of life, while appendectomy is unnecessary in the cases of normal appearance.

The significance of postoperative adjuvant therapy is uncertain in this rare disease. We found that the prognosis in these patients was remarkable, and postoperative adjuvant therapy did not improve OS, DSS, and RFS. Three of the eleven patients received chemotherapy or radiotherapy because of synchronous malignant tumors in other pathological types. Previous studies also suggested that surgery alone may be sufficient [2,7,16]. Therefore, in those without extra-ovarian spreading strumal carcinoid, postoperative adjuvant therapy should not be advocated. In the cases of patients presenting with other malignant tumors such as cervical, endometrial, and ovarian carcinoma, corresponding adjuvant therapy should be administered, including radiotherapy and chemotherapy [17–19]. Nonetheless, in patients without synchronous malignant tumor, adjuvant therapy may be preserved for those with recurrent or metastatic strumal carcinoid [6,7], since radical surgical resection may be unavailable in some situations such as multiple bones or liver metastasis. However, another dilemma is that strumal carcinoid may not be sensitive to chemotherapy [6], and radiotherapy may be more advisable for achieving disease remission [10].

We found that the Ki-67 index was fairly low in patients with strumal carcinoid, in keeping with previous research. Theurer et al. reported that 92.3% of patients (12 cases) had a Ki-67 proliferation index of less than 2%, and only one in 13 cases showed a Ki-67 proliferation index of 5% ^[[[4]]]^. In one patient with a Ki-67 index of 12%, she showed aggressive tumor behavior with multiple bones and breast metastasis, while a poor response to chemotherapy was also noted [6]. Moreover, the Ki-67 proliferation index is a reliable marker for predicting biological behavior and correlates with survival outcomes in gastro-intestinal NET and pulmonary carcinoid tumors [20,21]. In patients with pancreatic NET, a Ki-67 index cut-off of 2% to 10% was proposed to be a valid independent predictor [22–24]. In foregut NETs, Ki-67 is determined to assess the tumor grade, and three groups are defined: < 2%, between 3 and 20%, and > 20% [25]. Based on the former results and the classification of strumal carcinoid as midgut NET, it might be reasonable to propose a Ki-67 of 10% as a cut-off to predict biological behavior and survival. However, since a large proportion of patients lacked Ki-67 information, we failed to assess this.

This study has several limitations. Firstly, we did not evaluate the relationship between immunohistochemical and molecular characteristics and patient prognosis. Also, some patients in our database lacked comprehensive information on clinical manifestations. In addition, detailed descriptions of surgical procedures were unavailable in some literature. Lastly, only a small proportion of patients has a documented follow-up of more than 10 years, so the accurate long-term outcomes in this disease remain unresolved.

## Conclusion

Patients with ovarian strumal carcinoid have excellent prognoses irrespective of the surgical options, and the recurrence and disease-specific death rates were extremely low. USO without adjuvant therapy is preferred in patients with disease confined to the ovary, while conservative surgery with individualized postoperative adjuvant therapy is recommended in patients with metastatic lesions.

## Supplementary Information

**Additional file 1:** **Figure S1.** PRISMA flow diagram.

**Additional file 2:** **Figure S2.** The cumulative recurrent rate in this cohrot.

**Additional file 3:** **Figure S3.** The significantly different OS rate between age subgroups.

**Additional file 4:** **Table S1.** Database of our study.

**Additional file 5:** **Table S2.** The main clinical manifestations in patients with ovarian strumal carcinoid (*n* = 79).

**Additional file 6:** **Table S3.** Univariate and multivariate analysis of survival outcomes.

## Data Availability

All data generated or analyzed during this study are included in this published article and its supplementary information files. The datasets used and/or analyzed during the current study are available from the corresponding author upon reasonable request.

## Abbreviations

USO: Unilateral salpingo-oophorectomy
BSO: Bilateral salpingo-oophorectomy
H/BSO: Hysterectomy with bilateral salpingo-oophorectomy
NET: Neuroendocrine tumor
NSE: Neuron specific enolase
NA: Not applicable
