# Supplementary figures and images for: Clinical characteristics and survival outcomes in patients with ovarian strumal carcinoid
Source: BMC Cancer. 2022 Oct 24;22:1090. doi: 10.1186/s12885-022-10167-5 (PMC9594919; doi:10.1186/s12885-022-10167-5)

Figure S1. The detailed inclusion process according to the PRISMA flow diagram in our study.

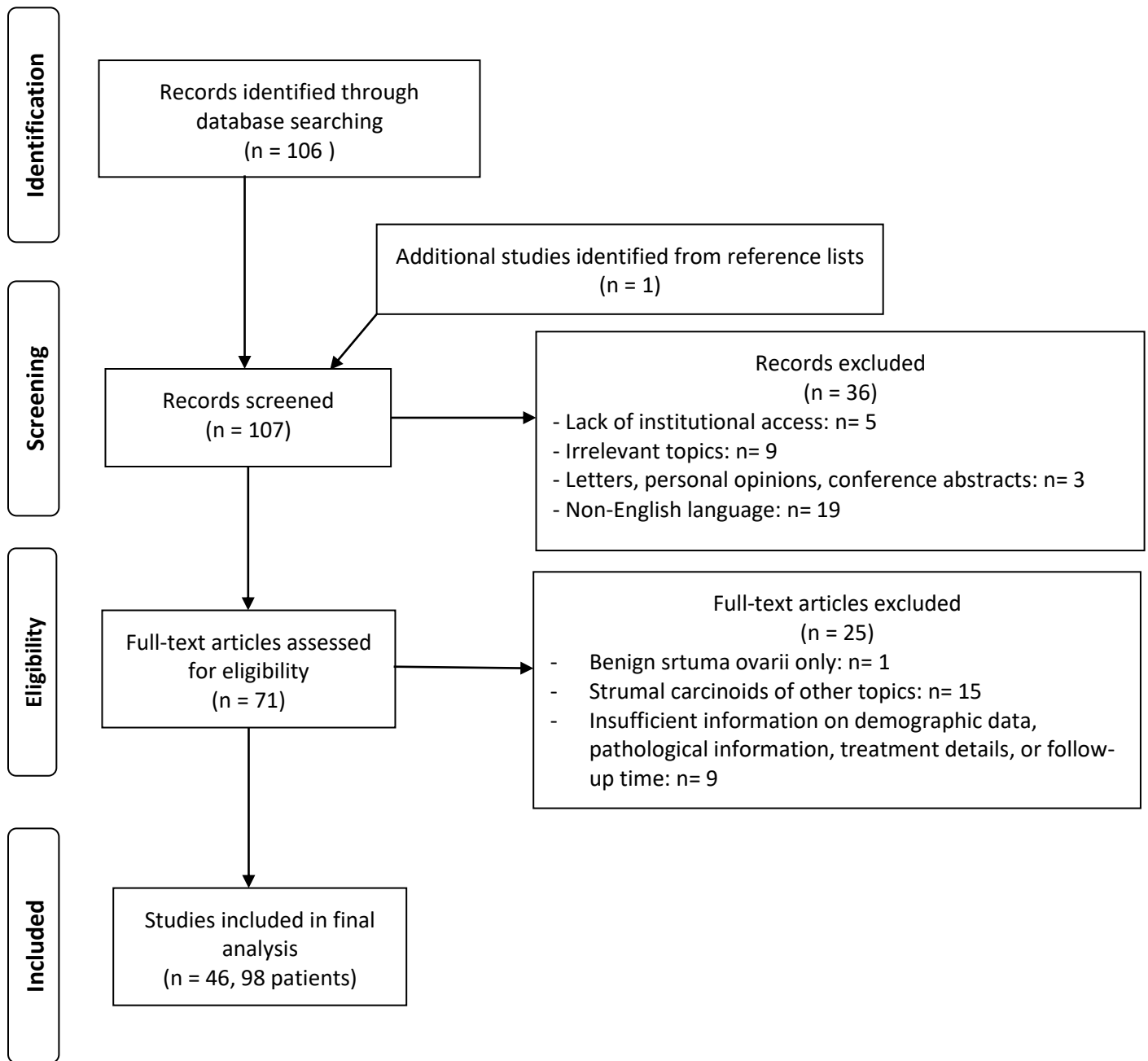

Supplement: Supplementary file 1 — Additional file 1: Figure S1. PRISMA flow diagram. [file 12885_2022_10167_MOESM1_ESM.pdf]

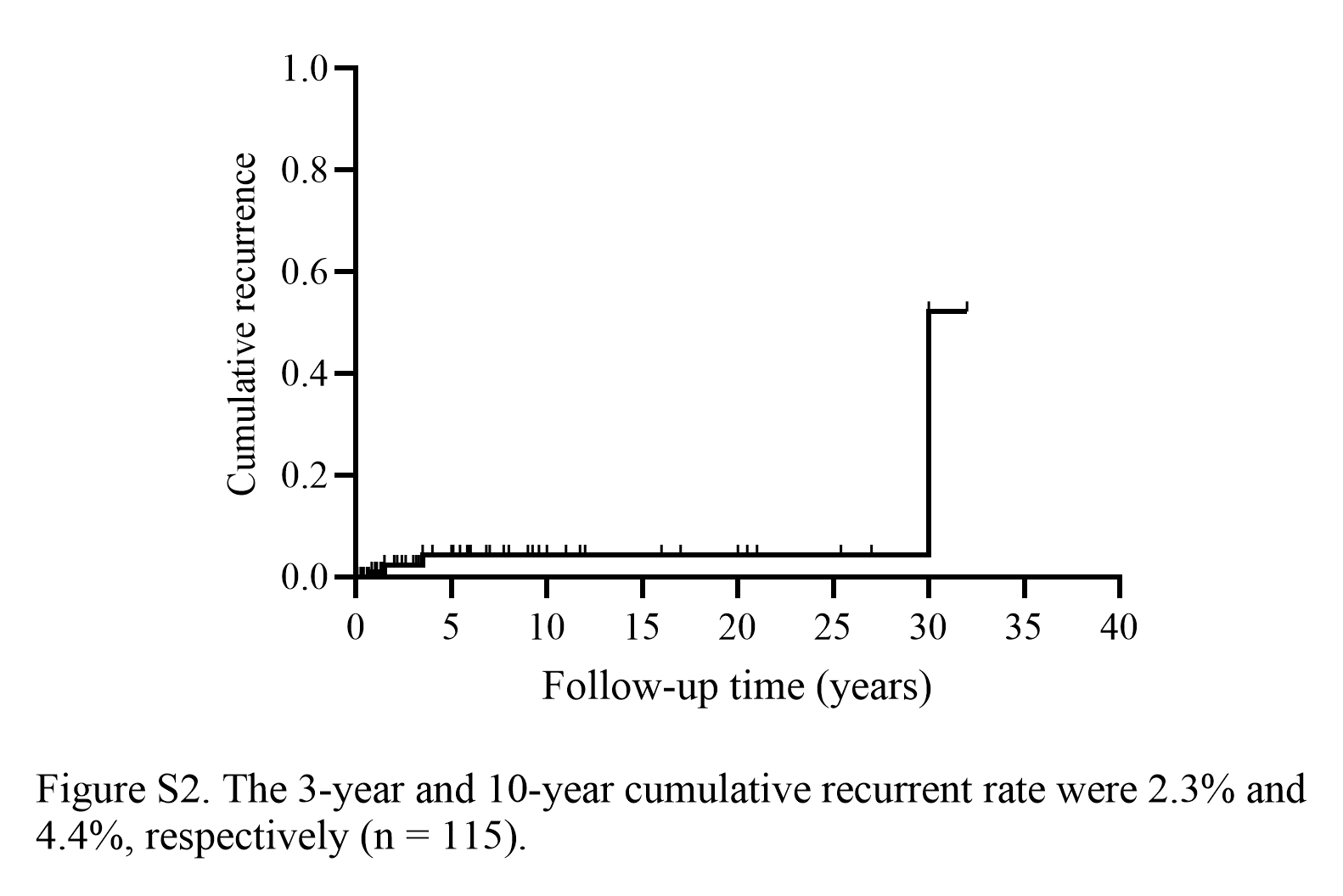

Supplement: Supplementary file 2 — Additional file 2: Figure S2. The cumulative recurrent rate in this cohrot. [file 12885_2022_10167_MOESM2_ESM.tif]

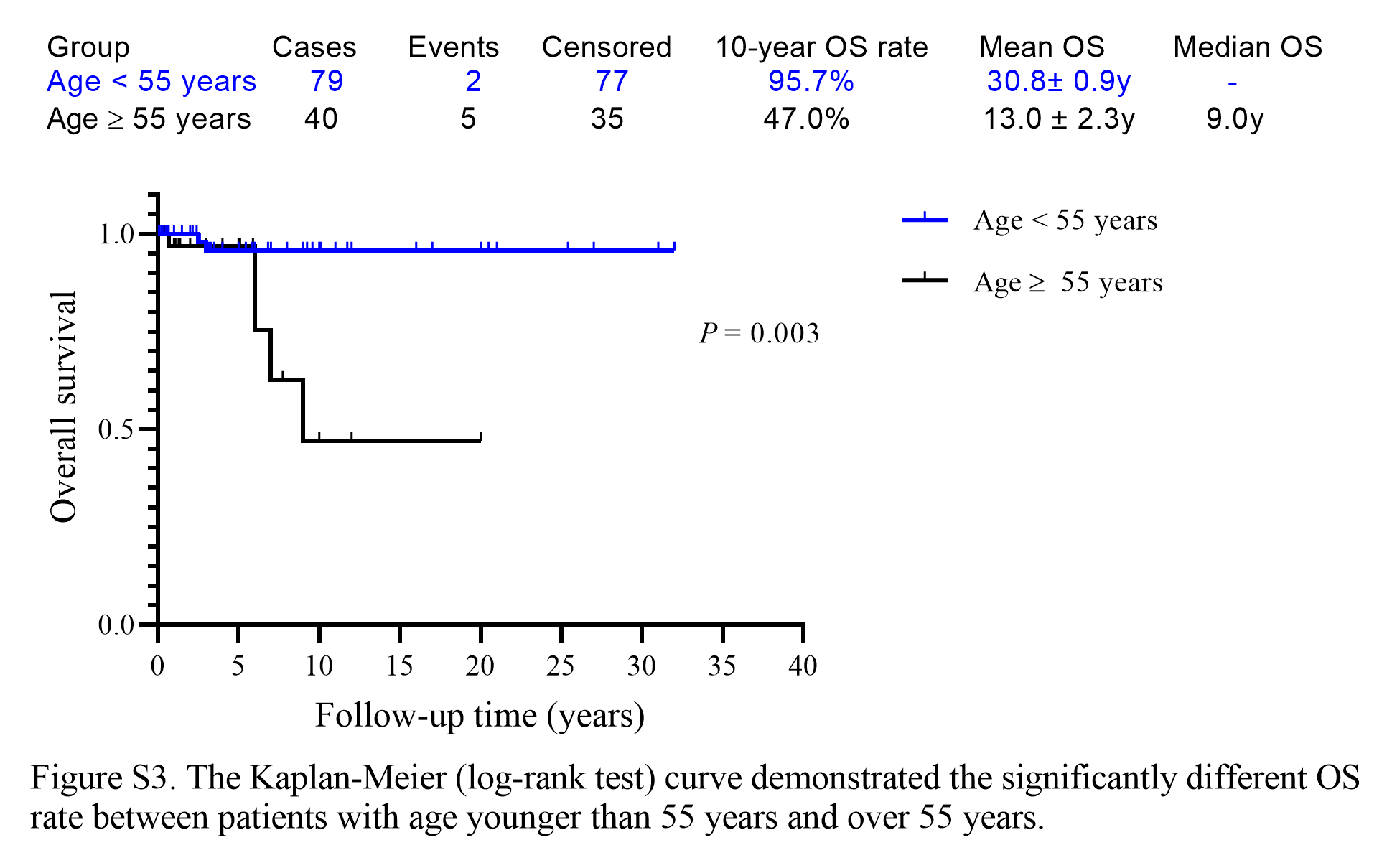

Supplement: Supplementary file 3 — Additional file 3: Figure S3. The significantly different OS rate between age subgroups. [file 12885_2022_10167_MOESM3_ESM.tif]
