# Supplementary material for: Clinical characteristics and survival outcomes in patients with ovarian strumal carcinoid
Source: BMC Cancer. 2022 Oct 24;22:1090. doi: 10.1186/s12885-022-10167-5 (PMC9594919; doi:10.1186/s12885-022-10167-5)
Supplement: Supplementary file 4 — Additional file 4: Table S1. Database of our study. [file 12885_2022_10167_MOESM4_ESM.docx]

Table S1. Database of our study.

| Reference | No. | Age  (y) | Non teratoma component | Clinical manifestations | Mass size (cm) | Surgery | Adjuvant  therapy | R | RFS  (y) | Ki-67 index | Recurrence  site | Treatment  In R | Results of follow-up |
| --- | --- | --- | --- | --- | --- | --- | --- | --- | --- | --- | --- | --- | --- |
| Cases in PUMCH | 1 | 35 | N | pelvic mass | 5.8 | USO (LSO) | N | N | 9.25 |  |  |  | NED at 9.25y |
|  | 2 | 56 | N | dysmenorrhea; menorrhagia | 6.7 | TH+BSO | N | N | 7.75 | <1% |  |  | NED at 7.75y |
|  | 3 | 32 | N | pelvic mass | 5.9 | Ovarian cystectomy | N | N | 6.83 |  |  |  | NED at 6.83y |
|  | 4 | 48 | N | pelvic mass | 3.3 | TH+BSO | N | N | 5.83 |  |  |  | NED at 5.83y |
|  | 5 | 47 | N | pelvic mass | 4.3 | TH+BSO | N | N | 3.16 | 3% |  |  | NED at 3.16y |
|  | 6 | 28 | N | pelvic mass | 5 | USO | N | N | 2.42 |  |  |  | NED at 2.42y |
|  | 7 | 53 | N | pelvic mass | 6 | USO | N | N | 2.17 | 1% |  |  | NED at 2.17y |
|  | 8 | 42 | N | pelvic mass | 10 | TH+BSO | N | N | 3.5 |  |  |  | NED at 3.5y |
|  | 9 | 44 | N | pelvic mass | 7.2 | USO (RSO) | N | N | 9.6 |  |  |  | NED at 9.6y |
|  | 10 | 39 | N | pelvic mass | 5 | USO (LSO) | N | N | 11.75 |  |  |  | NED at 11.75y |
|  | 11 | 38 | N | abdominal distention; ascites | 7.9 | USO (LSO) | N | N | 5.45 | 2% |  |  | NED at 5.45y |
|  | 12 | 30 | N | pelvic mass | 7 | USO (LSO) | N | N | 10.1 | 5% |  |  | NED at 10.1y |
|  | 13 | 46 | N | pelvic mass | 8.4 | Ovarian cystectomy | N | N | 0.3 | 5% |  |  | NED at 0.3y |
|  | 14 | 46 | N | pelvic mass | 2 | TH+LSO (RSO for pelvic mass previously) | N | N | 0.25 | 5% |  |  | NED at 0.25y |
|  | 15 | 65 | N | pelvic mass | 5.9 | TH+BSO+LN (pelvic and para-aortic) +appendectomy | N | N | 5.1 | <1% |  |  | NED at 5.1y |
|  | 16 | 47 | N | pelvic mass | 7.9 | TH+BSO | N | N | 3.3 | 1% |  |  | NED at 3.3y |
|  | 17 | 64 | adenocarcinoma | abdominal pain, pelvic mass | 18.4 | TH+BSO | N | N | 1.3 | 1% |  |  | NED at 1.3y |
|  | 18 | 64 | N | pelvic mass | 8 | TH + RSO + omentectomy; LSO for teratoma previously | N | N | 20 |  |  |  | NED at 20y |
|  | 19 | 30 | N | pelvic mass | 4 | Ovarian cystectomy | N | N | 1.5 | 2% |  |  | NED at 1.5y |
|  | 20 | 48 | N | abdominal distention | 10 | TH + BSO + omentectomy + LN (pelvic and para-aortic)+appendectomy | Chemotherapy | N | 25.4 |  |  |  | NED at 25.4y |
|  | 21 | 55 | N | pelvic mass | 7 | TH + BSO | N | N | 0.4 | 3% |  |  | NED at 0.4y |
| Dikman SH et al. (1971) | 22 | 53 | N | abdominal enlargement and hirsutism (masculinization) | 15 | TH+BSO | N | N | 7 |  |  |  | NED at 7y |
| Ranchod et al. (1976) | 23 | 45 | N | pelvic mass | 5.5 | TH+BSO | N | N | 0.67 |  |  |  | NED at 0.67y |
|  | 24 | 38 | N | left adnexal mass | 6 | TH+BSO | N | N | 2 |  |  |  | NED at 2y |
| Hart ea al. (1978) | 25 | 53 | N | postmeopause vaginal bleeding | 8 | TH+BSO | Radiotherapy | N | 20.5 |  |  |  | NED at 20.5y |
| Ueda et al. (1978) | 26 | 40 | N | slight abdominal pain | 6 | RSO (USO) | N | N | 2 |  |  |  | NED at 2y |
| Ancell B et al. (1979) | 27 | 67 | N | weakness, nausea, intermittent diarrhea | 15 | BSO | N | N | 0.5 |  |  |  | NED at 0.5y |
| Dayal Y et al. (1979) | 28 | 49 | N | irregular and heavy menstrual bleeding | 5.5 | TH+BSO | N | N | 5 |  |  |  | NED at 5y |
| Robboy SJ et al. (1980) | 29 | 27 | N |  | 6 | LO (USO) | N | N | 0.083 |  |  |  | NED at 1m |
|  | 30 | 48 | N |  | 4 | RO (USO) | N | N | 21 |  |  |  | NED at 21y |
|  | 31 | 58 | N |  | 8 | BSO | N | N | 5 |  |  |  | NED at 5y |
|  | 32 | 50 | N |  | 8 | TH+BSO | N | N | 9 |  |  |  | NED at 9y |
|  | 33 | 40 | Mucoid cystadenoma |  | 13 | TH+BSO | N | N | 0.083 |  |  |  | NED at 1m |
|  | 34 | 57 | N |  | 20 | BSO | N | N | 6 |  |  |  | Died of rheumatic heart disease at 6y |
|  | 35 | 57 | Mucoid cystadenoma |  | 7 | TH+BSO | Radiotherapy | N | 9 |  |  |  | Died of auto accident at 9y |
|  | 36 | 33 | N |  | 8 | BSO + appendectomy | N | N | 12 |  |  |  | NED at 12y |
|  | 37 | 71 | N |  | 14 | RO (USO)+appendectomy | N | N | 3 |  |  |  | NED at 3y |
|  | 38 | 32 | Foci brenner tumor |  | 8 | LSO (USO)+appendectomy | N | N | 10 |  |  |  | NED at 1oy |
|  | 39 | 47 | N |  | 13 | TH+BSO | N | N | 1 |  |  |  | NED at 1y |
|  | 40 | 59 | N |  | 17 | LSO(USO)+appendectomy | N | N | 0.42 |  |  |  | NED at 5m |
|  | 41 | 30 | N |  | 20 | TH+BSO | N | N | 3 |  |  |  | NED at 3y |
|  | 42 | 74 | N |  | 8 | TH+BSO | N | N | 3 |  |  |  | NED at 3y |
|  | 43 | 41 | N |  | 17 | TH+BSO | N | N | 10 |  |  |  | NED at 10y |
|  | 44 | 21 | N |  | 11 | LSO(USO)+appendectomy | N | N | 11 |  |  |  | NED at 11y |
|  | 45 | 58 | N |  | 9 | TH+BSO | N | N | 1 |  |  |  | NED at 1y |
|  | 46 | 68 |  |  | 15 | LO(USO) | Radiotherapy | N | 7 |  |  |  | Died of cerebrovascular accident at 7y |
|  | 47 | 41 | N |  | 2 | TH+BSO | N | N | 6 |  |  |  | NED at 6y |
|  | 48 | 38 | N | Constipation | 9 | TH+ BSO + appendectomy | N | N | 5 |  |  |  | NED at 5y |
|  | 49 | 53 | N |  | 12 | TH+BSO | N | N | 17 |  |  |  | NED at 17y |
|  | 50 | 74 | cystadenoma |  | 15 | TH+BSO | N | N | 0.67 |  |  |  | Died of intestinal necrosis at 8m |
|  | 51 | 53 | N | abdominal swelling | 16 | TH+BSO | N | Y | 1.5 |  | peritoneal cavity; liver | Laparotomy and thiotepa | DOD (recurrent strumal carcinoid) at 25y |
|  | 52 | 34 | N | ascites | 20 | LSO(USO) | Radiotherapy (X-ray) | N | 32 |  |  |  | NED at 32y |
|  | 53 | 52 | N | ascites, pleural effusion; abdominal swelling and pain | 26 | RSO (USO) | Radiotherapy (with Au 131 instilled into peritoneal cavity) | - |  |  | not CR;  liver and bowel metastasis initially |  | AWD at 12y |
|  | 54 | 42 | N |  |  | TH+BSO | Radiotherapy | N | 10 |  |  |  | NED at 10y |
|  | 55 | 40 | N |  | 4 | TH+LSO | N | N | 4 |  |  |  | NED at 4y |
|  | 56 | 30 | Insular carcinoid in appendix | Hirsutism; fatigue, abnormal menses | 5 | TH+LSO+appendectomy | N | N | 2 |  |  |  | NED at 2y |
|  | 57 | 33 | N |  | 4 | BO (BSO) | N | N | 2 |  |  |  | NED at 2y |
|  | 58 | 56 | N |  | 4 | TH+BSO | N | N | 4 |  |  |  | NED at 4y |
|  | 59 | 43 | N |  | 5 | LSO(USO) | N | N | 7 |  |  |  | NED at 7y |
|  | 60 | 54 | MSO |  | 6 | TH+BSO | N | N | 16 |  |  |  | NED at 16y |
|  | 61 | 49 | N | vaginal bleeding, ascites | 6 | TH+BSO | N | N | 12 |  |  |  | NED at 12y |
|  | 62 | 53 | N |  | 6 | RSO (USO) | N | N | 8 |  |  |  | NED at 08y |
|  | 63 | 40 | N |  | 8 | BSO | N | N | 5 |  |  |  | NED at 5y |
|  | 64 | 35 | N |  | 8 | TH+RSO | N | N | 27 |  |  |  | NED at 27y; |
|  | 65 | 38 | N |  | 10 | LO(USO)+appendectomy | N | N | 12 |  |  |  | NED at 12y |
|  | 66 | 54 | N |  | 11 | TH+BSO | N | N | 3 |  |  |  | Died of cirrhosis at 3y |
|  | 67 | 54 | MSO |  | 14 | BSO | N | N | 10 |  |  |  | NED at 10y |
|  | 68 | 52 | N |  | 15 | TH+BSO | N | N | 0.5 |  |  |  | NED at 0.5 |
|  | 69 | 77 | N |  | 16 | BSO | N | N | 3 |  |  |  | NED at 3y |
|  | 70 | 46 | N |  | 20 | LO(USO) | N | N | 1 |  |  |  | NED at 1y |
|  | 71 | 53 | N | Hirsutism | 20 | TH+ BSO + appendectomy | N | - |  |  | not CR; small bowel and descending colon metastasis |  | AWD at 10y |
| Ulbright TM et al. (1982) | 72 | 71 | Endometrial adenocarcinoma | postmenopausal vaginal bleeding | 13 | TH+BSO | Radiotherapy (for EC) | N | 10 |  |  |  | NED at 10y |
|  | 73 | 57 | N | flushing, diarrhea, and hypertension (carcinoid syndrome for 9 years) | 11 | TH+BSO | N | N | 0.33 |  |  |  | NED at 4m |
| Senteman MK et al. (1984) | 74 | 78 | N | epigastric pain and vomiting | 12 | LSO(USO) | N | N | 6 |  |  |  | NED at 6y |
| Morgan K et al. (1985) | 75 | 20 | N | abnormal vaginal discharge | 10 | LO (USO) | N | N | 20 |  |  |  | NED at 20y |
| Airo T et al. (1986） | 76 | 64 | N | large palpable mass | 12 | TH+BSO | N | N | 1.4 |  |  |  | NED at 1.4y |
| De Wilde et al. (1986) | 77 | 78 | N | pelvic mass | 14 | RO(USO) | N | N | 1 |  |  |  | NED at 1y |
| Steven DL et al. (1990) | 78 | 27 | N | hyperinsulinism |  | Ovarian cystectomy + distal pancreatectomy | N | N | 2 |  |  |  | NED at 2y |
| Motoyama T et al. (1992) | 79 | 53 | Serous cystadenoma | abdominal tumor, severe constipation | 10.5 | RSO (USO) | N | N | 3 |  |  |  | NED at 3y |
|  | 80 | 68 | N | abdominal tumor, severe constipation | 10 | TH+LSO | N | N | 3 |  |  |  | NED at 3y |
|  | 81 | 39 | Mucinous epithelium | abdominal tumor, severe constipation | 5 | LSO (USO) | N | N | 1 |  |  |  | NED at 1y |
| Armes JE et al. (1993) | 82 | 24 | N | amenorrhea and severe, acute, right-sided abdominal pain | 13 | RO (USO) + metastasectomy at 1st surgery. | Chemotherapy (PEB*3) and RAI*3 |  |  |  | Liver, peritoneum metastasis initially, not CR |  | AWD at 3m |
| Kataoka A et al. (1993) | 83 | 41 | N | slight abdominal pain | 4 | TH+BSO | N | N | 2 |  |  |  | NED at 2y |
| Ashton MA et al. (1995) | 84 | 59 | N | hypoglycemia, transit right hemiplegia; skin pigmentation | 13 | No | N |  |  |  | Not CR |  | Indirectly died of the disease at 6y |
| Matias G et al. (1995) | 85 | 51 | N | asymptomatic | 8.4 | RSO (USO) | N | N | 2 |  |  |  | NED at 2y |
| Takemori M et al. (1995) | 86 | 54 | Mucinous cystadenoma | lower abdominal pain | 11.5 | TH+BSO | N | N | 0.3 |  |  |  | NED at 0.3y |
| Davis KP et al. (1996) | 87 | 65 | N |  | 15 | TH+BSO+ appendectomy | N | N | 2 |  |  |  | NED at 2y |
|  | 88 | 49 | N |  | 8 | TH+BSO+ appendectomy | N | N | 2.5 |  |  |  | NED at 2.5 |
|  | 89 | 55 | N |  | 14 | TH+BSO+ appendectomy | N | N | 12 |  |  |  | NED at 12y |
|  | 90 | 22 | N |  | 4 | Ovarian cystectomy+ appendectomy | N | N | 5 |  |  |  | NED at 5y |
| Shigeta H et al. (1999) | 91 | 53 | Mucinous cystadenoma | progressive constipation | 16 | TH+BSO+LN+ omentectomy | Chemotherapy (cisplatin, Adriamycin, and cyclophosphamide for 3 cycles) | N | 2.6 |  |  |  | NED at 2.6y |
| Laurent B et al. (2001) | 92 | 68 | N | severe tricuspid insufficiency and 15kg weight loss (carcinoid heart disease) | 8 | Valve replacement and RSO (USO) | N | N | 3 |  |  |  | NED at 3y |
| Matsuda K et al. (2002) | 93 | 50 | N | persisting severe constipation | 20 | LSO (USO) | N | Y | 0.83 |  | liver | No, closed monitor | AWD at 3y |
| Seok MK et al. (2003) | 94 | 62 | Mucinous adenocarcinoma; SCC in cervix | lower abdominal pain | 7 | TH+BSO | Chemotherapy (taxol + cisplatin) and EBRT | N | 1.1 |  |  |  | NED at 1.1y |
| Kouichiro K et al. (2007) | 95 | 47 | N | persisting severe constipation | 12 | TH+BSO+pelvic and para-aortic LN sampling, omentectomy, and appendectomy | Chemotherapy (paclitaxel + carboplatin for 3 cycles) | N | 1.5 |  |  |  | NED at 1.5y |
| Gorin I et al. (2008) | 96 | 63 | N | hyperandrogenemia (hirsutism) | 14 | LSO (USO) | N | N | 1 |  |  |  | NED at 1y |
| Pelosi G et al. (2008) | 97 | 69 | MSO (PTC) | pelvic mass | 21 | LO (USO) | N | N | 1 |  |  |  | NED at 1y |
| Kachhawa (2011) | 98 | 60 | N | severe progressive constipation | 8 | TH+BSO | N | N | 0.5 |  |  |  | NED at 0.5y |
| Takumi et al. (2010) | 99 | 34 | N | lower abdominal distension | 14 | TH+BSO+omentectomy+appendecomy+LN (pelvic and para-aortic) | N | Y | 3.5 | 12% | multiple bone and breast | Chemotherapy (paclitaxel + carboplatin, etoposide + cisplatin) and radiotherapy | AWD at 3.5y |
| Takatori E et al. (2012) | 100 | 48 | N | severe constipation | 6 | RSO (USO) | N | N | 1.5 |  |  |  | NED at 1.5y |
| Hayashi T et al. (2012) | 101 | 45 | N | hypermenorrhea and anemia-related symptoms | 3 | TH+BSO | N | N | 1 | 3% |  |  | NED at 1y |
| Hinshaw et al. (2012) | 102 | 74 | MSO; mucinous adenocarcinoma | postoperative edema | 8.5 | TH+BSO+omentectomt+pelvic and para-aortic LN | N | N | 0.5 |  |  |  | NED at 0.5y |
| Bohara S et al. (2013) | 103 | 60 | Mucinous cystadenoma | progressive enlarged pelvic mass | 24 | RSO (USO) | N | N | 0.25 |  |  |  | NED at 0.25y |
| Sulaiman S et al. (2013) | 104 | 30 | N | severe constipation; hirsutism and clitoromegaly | 11.4 | RO (USO) | N | N | 0.3 |  |  |  | NED at 0.3y |
| Muller KE et al. (2015) | 105 | 34 | N | severe constipation, left-sided abdominal patin, nausea, decresed appetite | 9 | RSO (USO)+LN+omental biopsy | N | N | 0.13 |  |  |  | NED at 0.13y |
| Motoyama T et al. (1992) | 106 | 53 | Serous cystadenoma | abdominal tumor, severe constipation | 10.5 | RSO (USO) | N | N | 3 |  |  |  | NED at 3y |
|  | 107 | 68 | N | abdominal tumor, severe constipation | 10 | TH+LSO | N | N | 3 |  |  |  | NED at 3y |
|  | 108 | 39 | N | abdominal tumor, severe constipation | 5 | LSO （USO) | N | N | 1 |  |  |  | NED at 1y |
| Quinonez E et al. (2015) | 109 | 58 | N | increasing abdominal pain, distension, and weight loss | 18 | TH+BSO+omentectomy+appendectomy (debulking) | N | N | 0.25 |  | Omentum and appendix metastasis initially |  | NED at 0.25y |
| Tarcoveanu E et al. (2015) | 110 | 55 | Cystic lymphangioma | lower abdominal pain | 7.1 | LSO (USO)+ cystectomy | N | N | 1 | <1% |  |  | NED at 1y |
| Kim et al. (2016) | 111 | 39 | Mucinous carcinoid | low back pain | 9.7 | RSO (USO) | N | N | 0.6 | <1% |  |  | NED at 0.6y |
| Ciobanu et al. (2017) | 112 | 24 | Mucinous cystadenoma | mild diffuse pelvic pain |  | RSO (USO) | N | N | 1 | <1% |  |  | NED at 1y |
| Noh HK et al. (2017) | 113 | 64 | N | persisting severe constipation | 6.3 | TH+BSO+omentectomy (debulking) | N | N | 1.4 | 10% |  |  | NED at 1.4y |
| Antovska VS et al. (2018) | 114 | 59 | N | pelvic mass | 6 | TH+BSO+omentectomy and peritoneal washing sampling, biopsy | N | N | 2 |  |  |  | NED at 2y |
| Ishida M et al. (2018) | 115 | 68 | N | pelvic mass | 5 | BSO | N | N | 0.33 |  |  |  | NED at 4m |
| Borghese M et al. (2018) |  | 31 | N | pelvic mass | 1 | left ovarian cystectomy | N | Y | 30 | <2% | contralateral ovary and para-aortic lymph nodes | RSO and lymph nodes resection | NED at 31y |
| Cagino K et al. (2020) |  | 48 | MSO (PTC) | sharp lower abdominal pain and abnormal uterine bleeding | 10 | TH+BSO | N | N | 0.5 |  |  |  | NED at 0.5y |
| Yan F et al. (2021) |  | 55 | N | pelvic mass | 8.6 | RSO (USO) | N | N | 5.91 | 1-2% |  |  | NED at 71m |
| Armes JE et al. (1993) |  | 45 | Mucinous cystadenoma | pelvic mass | 12 | RSO (USO) | N | N | 1 | 1-2% |  |  | NED at 1y |

Abbreviations: MSO, malignant struma ovarii; PTC, papillary thyroid carcinoma; USO, unilateral salpingo-oophorectomy; BSO, bilateral salpingo-oophorectomy; TH, total hysterectomy; LN, lymph nodes resection; RAI, radioiodine therapy; EBRT, external beam radiotherapy; NED, no evidence of disease; AWD, alive with disease; DOD, die of the disease; N, no; Y, yes; NA, not applicable; R, recurrence; RFS, recurrent free survival.
