# Supplementary material for: Clinical characteristics and survival outcomes in patients with ovarian strumal carcinoid
Source: BMC Cancer. 2022 Oct 24;22:1090. doi: 10.1186/s12885-022-10167-5 (PMC9594919; doi:10.1186/s12885-022-10167-5)
Supplement: Supplementary file 5 — Additional file 5: Table S2. The main clinical manifestations in patients with ovarian strumal carcinoid (n = 79). [file 12885_2022_10167_MOESM5_ESM.docx]

Table S2. The main clinical manifestations in patients with ovarian strumal carcinoid (n = 79).

| Clinical manifestations | N = 79 |
| --- | --- |
| Pelvic mass | 30 (38.0%) |
| Abdominal pain/discomfort | 16 (20.2%) |
| Constipation | 15 (19.0%) |
| Hirsutism | 5 (6.3%) |
| Others | 13 (16.5%) |
| Vaginal bleeding/AUB | 7 |
| Hyperinsulinism/hypoglycemia | 2 |
| Carcinoid heart disease | 1 |
| Nausea, diarrhea | 1 |
| ascites | 2 |

Abbreviations: AUB, abnormal uterine bleeding.
