# Supplementary material for: Clinical characteristics and survival outcomes in patients with ovarian strumal carcinoid
Source: BMC Cancer. 2022 Oct 24;22:1090. doi: 10.1186/s12885-022-10167-5 (PMC9594919; doi:10.1186/s12885-022-10167-5)
Supplement: Supplementary file 6 — Additional file 6: Table S3. Univariate and multivariate analysis of survival outcomes. [file 12885_2022_10167_MOESM6_ESM.docx]

**Table S3a** Univariate and multivariate analysis of OS

| Factors | N |  | Univariate analysis | |  | Multivariate cox regression analysis | | | |
| --- | --- | --- | --- | --- | --- | --- | --- | --- | --- |
|  |  | Mean survival(y) | 10-year survival rate | p |  | OR | (95% CI) | | p |
| Age (<55/>=55, years) ^a, *^ | 79/40 | 30.8/13.0 | 95.6%/38.7% | 0.003 |  | 7.988 | | 1.519-42.004 | 0.014 |
| Metastasis (No/Yes) | 115/4 | -/- | 84.3%/100% | 0.560 |  |  | | | |
| Non teratoma component (No/Yes) | 97/22 | 28.7/13.7 | 87.5%/75.0% | 0.266 |  |  | | | |
| Tumor size (<10/>=10, cm) ^a, *^ | 64/52 | 29.5/25.0 | 93.3%/74.5% | 0.028 |  |  | | | |
| Surgical options (conservative/radical) | 49/69 | 30.3/23.4 | 93.3%/83.1% | 0.203 |  |  | | | |
| Adjuvant therapy (Yes/No) | 12/107 | 26.0/28.1 | 75.0%/89.1% | 0.478 |  |  | | | |

a, Factors applied to multivariate analysis; -, Not available; *, p < 0.05

**Table S3b** Univariate and multivariate analysis of RFS

| Factors | N | Univariate analysis | |  | Multivariate cox regression analysis | | | |
| --- | --- | --- | --- | --- | --- | --- | --- | --- |
|  |  | 10-year RFS rate | p |  | OR | (95% CI) | | p |
| Age (<55/>=55, years) | 76/39 | 94.0%/100% | 0.288 |  |  | |  |  |
| Metastasis (No/Yes) | 114/1 | 95.6%/100% | - |  |  | | | |
| Non teratoma component (No/Yes) | 93/22 | 95.0%/100% | 0.508 |  |  | | | |
| Tumor size (<10/>=10, cm) | 64/48 | 100%/88.0% | 0.157 |  |  | | | |
| Surgical options (conservative/radical) | 47/68 | 97.5%/94.5% | 0.804 |  |  | | | |
| Adjuvant therapy (Yes/No) | 10/105 | 100%/95.0% | 0.251 |  |  | | | |

a, Factors applied to multivariate analysis; -, Not available; *, p < 0.05.

**Table S3c** Univariate and multivariate analysis of DSS

| Factors | N | Univariate analysis | |  | Multivariate cox regression analysis | | | | |  |
| --- | --- | --- | --- | --- | --- | --- | --- | --- | --- | --- |
|  |  | 10-year survival rate | p |  | OR | | (95% CI) | | p | |
| Age (<55/>=55, years) | 79/40 | 97.9%/88.9% | 0.439 |  |  |  | |  | |  |
| Metastasis (No/Yes) | 115/4 | 95.7%/100% | 0.769 |  |  | | | | |  |
| Non teratoma component (No/Yes) | 97/22 | 95.3%/100% | 0.592 |  |  | | | | |  |
| Tumor size (<10/>=10, cm) | 64/52 | 100%/91.0% | 0.123 |  |  | | | | |  |
| Surgical options (conservative/ radical) | 49/69 | 100%/97.6% | 0.435 |  |  | | | | |  |
| Adjuvant therapy (Yes/No) | 12/107 | 95.0%/100% | 0.516 |  |  | | | | |  |

a, Factors applied to multivariate analysis; -, Not available; *, p < 0.05.

Abbreviations: OS, overall survival; RFS, recurrent-free survival; DSS, disease-specific survival.
